# Supplementary material for: Are carbon emissions trading and green financial instruments synergistic? -Comprehensive quantitative research based on content analysis
Source: PLoS One. 2024 Mar 7;19(3):e0298601. doi: 10.1371/journal.pone.0298601 (PMC10919601; doi:10.1371/journal.pone.0298601)
Supplement: S1 Data — (ZIP) [file pone.0298601.s001.zip › Data/地级市Z_F工作报告.zip!/╔╜╢1⁄2/╝├─■╩╨/└┤╫╘╣1⁄2╓┌║┼í╛┬φ┐╦╩2╛▌═°í┐_2012.docx]

各位代表：

现在，我代表济宁市人民政府向大会报告工作，请予审议，并请市政协委员和其他列席会议的同志提出意见。

一、2008年以来工作回顾

过去的四年很不寻常。面对复杂形势和艰巨任务，全市上下在中共济宁市委的坚强领导下，坚持科学发展跨越发展总基调，突出重点、突出特色、突出创新，有效克服国内外经济环境变化带来的不利影响，扎实做好保增长、惠民生、促和谐各项工作，圆满完成市十五届人大历次会议确定的年度任务，“十一五”规划目标顺利实现，确保了“十二五”发展的良好开局。2011年全市地区生产总值达到2896.7亿元，比2007年增长59.4%；规模以上工业主营业务收入4276亿元、是2007年的2.2倍；地方财政收入207亿元，四年翻了一番；固定资产投资累计4879亿元，年均递增24%；金融机构年末存款余额2612.5亿元、贷款余额1613.6亿元，分别比2007年增加1443.6亿元和883.2亿元；城镇居民人均可支配收入19215元，农民人均纯收入8712元，比2007年增长57.8%和65.3%。我市综合实力跃上新的台阶，在富民强市征程上迈出坚实步伐。

（一）转型发展步伐加快。煤电产业居主导地位的格局开始扭转，优势传统产业蓬勃发展，制造业增加值占规模以上工业的比重上升到56%，四大千亿级产业集群总量突破3000亿，销售收入过百亿企业增加到7家。战略性新兴产业和高新技术产业快速成长，建成国家级光电产业基地，济宁高新区晋升为国家级高新区，全市高新技术产业产值751亿元、占规模以上工业的19.4%。创出15个中国驰名商标、59个省著名商标和159个全国全省名牌产品。科技、金融、物流等新兴服务业增势强劲，鲁南工程技术研究院等一批高层次研发机构相继落成，兴业等4家商业银行和豪德物流等一批知名企业入驻济宁，宝相寺景区等一批文化旅游重点项目投入运营，全市旅游社会总收入286.4亿元、是2007年的2.3倍。农村六大重点工程成效显著，粮食实现连续8年增产，建成农业标准化基地350万亩，金乡大蒜、邹城食用菌、微山渔湖产品、梁山鲁西黄牛等品牌效益更加凸显，市级以上农业龙头企业和专业合作组织发展到767家、2095家。

（二）改革开放纵深推进。国有企业改革取得突破性进展，50家市属困难国有企业完成改制和破产重组。行政管理体制改革扎实有效，共压减行政审批事项167个，成立市公共资源交易平台，工程招投标、政府采购、土地出让、产权交易实现全程网上监管，涉企收费“一费制”改革经验在全国推广。投融资体制改革迈出新步伐，市县两级投融资平台累计融资353亿元。农村集体林权制度主体改革全面完成，“镇园合一”管理体制逐步到位。民营经济发展活力增强、贡献份额增加，实现增加值、上交税金占全市的比重达到65%、61%。对外开放领域进一步拓展，境内外招商活动成果丰硕，四年引进市外国内资金1562.1亿元，直接利用外资由2007年的4.2亿美元增加到7.3亿美元。39家世界500强企业落户济宁。外贸进出口总额57.5亿美元，是2007年的2倍，13家企业在境外投资办厂。兖矿集团、如意科技等企业相继走出国门，在跨国并购和国际市场竞争中取得突破性成就。

（三）城乡面貌变化巨大。城镇化追赶战略加快推进，“两区三市”全部实现镇街建制。“一城四区”城市框架全面拉开，北湖新城展示靓丽形象，济北新区承载能力迅速提升，东部科技新城服务功能显著增强，运河生态经济区加紧建设，中心城区建成面积由2007年的60平方公里增加到117.5平方公里，人口由60万人增加到102.4万人。济兖邹曲嘉五大板块加速融合，太白楼路东西跨河大桥及延伸线、临菏路济宁段建成通车，济宁机场开通8条航线，京沪高铁曲阜站竣工运营，京杭运河济宁至韩庄段三级航道、森达美跃进港区等投入使用。南水北调东线境内工程全面开工，治淮南下、病险水库除险加固工程基本完成。县域老城改造、新城建设力度加大、面貌焕然一新，小城镇开发特色凸显，建成332个新型农村社区，城乡环卫一体化逐步形成。新建改建农村公路3865公里，全市所有乡镇和99.6%的行政村实现路面硬化。

（四）生态环境明显改善。“十一五”节能减排目标如期完成，节能减排两项工作均在全省获奖。探索出“治用保防控”结合的治污新模式，完成211个工业点源深度治理项目，淘汰255家企业落后产能，97家重点排污企业实现稳定达标排放。大气污染防治措施到位，在全省率先完成燃煤机组脱硫改造任务，化工发酵企业异味污染得到遏制，秸秆禁烧形成长效机制。持续开展荒山治理，东部山区再现青山绿荫景观，全市森林覆盖率达到28%。启动环南四湖大生态带建设，建成和修复人工湿地16万亩，微山湖湿地公园晋升为国家湿地公园，治理采煤塌陷地5.8万亩。

（五）人民群众得到更多实惠。各项惠民富民政策全面落实，连年承诺为民所办10件实事如期兑现。市级财政累计民生投入71.4亿元，年均增长22.7%。四年新增城镇就业38.1万人，农村劳动力转移就业37.2万人，高校毕业生就业14.8万人，城镇登记失业率稳定在4%以内。新农合、新农保和城镇居民养老保险实现全覆盖，全市60岁以上城乡居民都能按月领到养老金。医改五项重点工作圆满完成，基本药物制度全面实施，医疗保险“一证通”和“先看病后付费”就诊模式在全市推广，完成143处乡镇卫生院、149处社区卫生服务机构和3689个村卫生室的规范化建设，群众看病难、看病贵问题明显缓解。连续调高企业最低工资和退休职工养老、城乡低保及“五保”供养标准，残疾人生活补助、康复救助制度及时落实。6.3万户城市居民得到住房保障，新建农村住房37.1万户、改造农村危房1.4万户，城乡群众住房条件不断改善。

（六）社会事业长足进步。城乡免费义务教育全面实现，优质教育资源配置和均衡发展取得积极进展。四年加固重建校舍184万平方米，县乡办学条件普遍改善，省级规范化学校增加到124所。文化建设领域拓展、成效显著，建成曲阜国家级文化产业示范园和6处省级以上文化产业示范基地，启动孔孟文化遗产地保护世行贷款项目，国际孔子文化节、中国专博会影响力持续提升，成功举办4届“世界儒学大会”及首届“尼山世界文明论坛”，一批文艺精品在全国、全省获奖。全民健身公共设施不断完善，建成3000个村级农民体育健身工程，赢得第23届省运会承办权，我市运动员在亚运会上夺取两枚金牌。科技事业成绩斐然，获得省级以上科技进步奖105项，授权专利1.37万件，被评为全国科技进步先进市。顺利完成第六次人口普查，人口自然增长率稳定在6‰左右。扎实开展食品药品安全专项整治，工商、物价和质量技术监管全面加强。安全生产形势持续稳定好转。援建北川项目提前竣工，新一轮援疆工作有效推进。国防动员、人民防空、民兵预备役和国家安全工作成效明显，连续六次荣获“全国双拥模范城”称号。广播电视、新闻出版、民族宗教、住房公积金、外事侨务和对台工作取得新成绩，地震、气象、水文、档案、史志、文博、红十字、慈善、老龄和妇女儿童等项事业健康发展。

（七）民主法制和精神文明建设切实加强。自觉依照法定权限和程序行使职权，规范优化政务运行流程，完善行政审批中心运作机制，积极推进行政复议委员会改革，依法行政水平和服务效能得到提高。及时向人大报告工作、向政协通报情况，人大代表建议和政协提案办复率、满意率分别达到100%、98.2%。顺利完成第十届村民委员会换届直选。全面落实政务公开，建成统一的政务外网平台，政府工作透明度、开放度进一步增强。行政问责、效能监察和审计监管力度加大，政府公信力、执行力持续提升。深入推进精神文明创建、公民道德建设和未成年人思想道德建设，公民文明素质和社会文明程度普遍提高。5个单位获得全国文明单位称号，我市跻身省级文明城市行列。“平安济宁”建设扎实推进，社会管理创新不断加强，矛盾排查和群众利益诉求渠道更加畅通，处置公共突发事件能力进一步提高，联手创建鲁苏边界和谐维稳机制更加完善，我市被评为“全国社会治安综合治理优秀市”。

各位代表！过去的四年是我们战胜困难挑战、开拓创新奋进、力促转型发展阔步前行的四年，是全市经济持续快速增长、城乡面貌显著改观、城乡群众受益最多的四年，是各项社会事业统筹协调推进、和谐稳定局面不断巩固发展的四年。四年来，我们把加快发展作为最大任务，始终坚定紧抓第一要务的信心，强化又好又快的导向，创新克难攻坚的举措，及早把加快发展的突破口聚焦在转方式调结构上，全力推进经济战略转型，逐步打开资源型城市转型发展、跨越崛起的新路径，为构筑济宁长远发展的战略优势奠定了坚实基础。我们把项目带动作为重要抓手，抢抓国内外产业资本加速转移流动的难得机遇，创新方式、拓展领域，展开高层对接，提升招商成效，一批投资规模大、带动作用强的重大产业项目、文化旅游项目和城市综合体项目落地建设，开创出依靠招大引强推动产业升级、促进城市开发、繁荣现代服务业的新天地。我们把创新驱动作为核心战略，制定吸引力强的优惠政策，依据高新区展开“人才特区”建设试点，高标准建设科技研发平台，相继引进一批高层次专家和领军型创新人才，同步推进产学研和科技金融紧密合作，我市加快发展的人才保障和智力支撑能力日趋强劲。我们把保障改善民生作为最高追求，扎实推进创业富民、就业惠民、社保安民，每年兴办一批民生实事项目和惠民系列工程，大幅增加民生和社会事业投入，切实做到发展为了人民、发展依靠人民、发展成果由人民共享。我们把办好大事硬事作为工作突破口，发挥整体优势，选准一个时期、每个年度的重大事项，凝聚合力集中推进，办成了一批包括城区区划调整、重大基础设施建设在内的大事硬事好事。特别是2011年，代表山东省接受国家淮河流域治污考核，并再次获得第一名；“大干100天”如期建成京沪高铁曲阜站，被评为沿线六大精品工程之一；高水平举办中国文化遗产日主场城市活动，创出主场城市活动新标杆；全方位实施城区绿地、大气环境和水质保护综合整治，顺利通过国家园林城市考核验收。这些探索实践和成功做法，不仅确保了过去改革建设各项工作的有效开展，也必将成为指导和推动今后一个时期更好更快发展的宝贵财富。

各位代表！四年的历程波澜壮阔，辉煌的成就令人鼓舞。这是省委、省政府和市委正确领导的结果，是市人大、市政协和社会各界监督支持的结果，是历届政府班子继往开来、接力奋斗的结果，是全市人民团结实干、无私奉献的结果。在此，我代表济宁市人民政府，向全市工人、农民、知识分子和干部，向各级人大代表、政协委员，各民主党派、工商联、各人民团体和各界人士，向中国人民解放军驻济部队指战员、武警官兵和公安干警，向中央、省驻济单位，向支持参与济宁建设发展的港澳台同胞、海外侨胞和国际友人，表示衷心的感谢和崇高的敬意！

我们也清醒地看到，济宁的发展还面临许多困难和问题，政府工作中也存在不少差距和不足。主要是：经济结构性矛盾依然突出，产业层次不高、服务业发展滞后；人均占有和城镇化水平偏低，自主创新能力不够强，节能减排压力较大；县域经济还比较薄弱，发展不平衡问题尤为突出；农民增收渠道较窄，部分群众生活仍较困难；社会治安、城市管理和公共服务还需加强；政府职能转变不快，有些干部思想观念、能力作风还有待改进和提高。我们必须高度重视存在问题，采取有效措施认真加以解决。

二、新一届政府面临的形势和任务

今后五年是我市科学发展跨越发展的重要时期，也是加快建成全面小康社会的攻坚阶段。以党的十八大召开为标志，我国将开启改革开放和现代化建设新征程，推进科学发展、增进民生福祉、促进社会和谐的举措更加有力。新一轮产业和科技革命提速推进，发达国家和地区发展方式转变、产业资本转移步伐加快，大跨度吸纳集聚先进要素，借助外力跨越崛起的条件更加便利。我市综合实力不断攀升，交通、水利、能源等基础设施支撑体系日益完善，一批重大产业项目陆续进入收获期，加快发展的持续性稳定性更加凸显。市十二次党代会确定了今后五年的宏伟蓝图，换届后的各级领导班子年富力强、思路开阔，干事创业的热情更加高涨。这些都为我们在更高起点上推进科学发展跨越发展带来难得机遇、增添新的动力。同时也要看到，国际金融危机的影响短期内难以消除，世界经济复苏步伐缓慢，我国经济运行中也遇到一些新情况新变化，我市周边地区发展势头迅猛，竞争压力增大。我们务必增强责任意识和忧患意识，以更加宽阔的视野审时度势，以时不我待的斗志抢抓机遇，负重奋进，开拓创新，努力夺取经济文化强市建设新胜利。

今后五年政府工作总体要求是：高举中国特色社会主义伟大旗帜，以邓小平理论和“三个代表”重要思想为指导，全面落实市十二次党代会既定部署，坚持科学发展跨越发展总基调，以加快转变经济发展方式为主线，以富民惠民为首要任务，大力实施工业强市、创新驱动、开放带动、可持续发展战略，强力突破工业化、城市化两大重点，更加注重强化农业基础和发展现代农业，更加注重保障改善民生，更加注重加强社会管理创新，更加注重维护社会和谐稳定，加快推进经济跨越发展和社会全面进步，确保综合实力进入全省第一方阵，争当淮海经济区排头兵，全面建成惠及全市人民的小康社会。

今后五年的奋斗目标是：全市总体建成全面小康社会，强县向更高水平的小康迈进，中等县全面达小康，薄弱县基本实现全面小康，人民群众普遍过上经济殷实、文化丰富、生态宜居、安定和谐的小康生活。

──综合实力显著增强。经济总量、均量、质量持续提升，地区生产总值、地方财政收入实现翻番，主要人均指标赶上全省平均水平。区域均衡发展取得重大进展，产业结构更加优化，经济整体素质和综合竞争实力大幅提高。

──人民生活显著改善。全市人民共同富裕步伐加快，城乡居民收入实现倍增，家庭财富尤其是中低收入家庭财富大幅增加。大学生和困难群体实现充分就业，人民群众普遍享受良好教育，养老、医疗和失业保险等社会保障体系更加健全，城乡群众生活质量和幸福指数明显提高。

──城乡面貌显著变化。城乡一体化进程不断加快，城镇化率达到全省平均水平，中心城区辐射带动能力明显增强，都市区实现同城发展，县城和小城镇功能形象进一步提升，新农村建设取得实质性突破。市域大生态体系基本形成，南四湖水质全面稳定达标，各类资源集约节约利用，建成国家生态园林城市。

──社会文明显著提升。各项社会事业全面发展，全民思想道德素质、科学文化素质和健康素质普遍提升，群众权益和公平正义得到切实保障，社会管理科学化水平明显提高，社会安定有序、文明和谐、充满活力，孔孟之乡知礼厚德、诚信守法、文明谦和、开放包容的形象充分彰显，建成全国文明城市。

围绕实现上述目标，政府工作的主要任务是：强化“三大支撑”、构筑“三大高地”、打造“三大环境”，为全市经济高位求进、跨越赶超奠定坚实基础、提供有力保障。

一是强化现代产业、城镇化和县域经济“三大支撑”。以高端高质高效为引领，更大力度推进经济战略转型，快速膨胀先进制造业，培植更多优势产业集群，壮大高新技术产业和战略性新兴产业规模，整体提升工业经济实力。坚持扩大总量和优化结构并举，强化政策扶持、载体建设，不断拓展领域、创新业态，大幅提高服务业发展水平和比重。加快转变农业发展方式，优化种养结构，发展高效农业，加速向规模化布局、标准化生产、产业化经营迈进。到2016年，全市工业销售收入突破万亿规模，建成国内重要的装备制造业、国家级煤化工、纺织新材料和战略性新兴产业“四大基地”，服务业增加值占GDP的比重提高到45%以上，基本形成以高新技术产业为先导，先进制造业和现代服务业为支撑，现代农业为基础的现代产业体系。以持续开展“城市建设管理年”活动为牵动，加快城镇化追赶步伐，按照“一城四区”发展格局，进一步拉开框架、扩大规模、优化布局、提升形象，五年内中心城区建成区面积达到150平方公里、人口达到150万。坚持功能互补、资源共享、相向发展，实质性推进济兖邹曲嘉融合对接，适时启动城际轨道交通，加快形成组团发展格局。坚定不移实施“县域经济倍增计划”，加大招大引强、项目带动和园区建设力度，加快培育特色主导产业，促使立县立乡大企业加速崛起，以工业振兴壮大县级财力，带动县域经济跨越发展。用3－5年时间，12个县市区固定资产投资、规模以上工业增加值、地方财政收入全部实现翻番，地方财政收入全部过10亿元、半数超过20亿元。

二是构筑自主创新、人才队伍和文化发展“三大高地”。深入实施科技创新系列工程，健全完善高端研发平台，纵深推进产学研和科技金融紧密结合，突破共性关键技术，培育更多自主知识产权、自主品牌和创新型企业，建设知识产权强市，打响“济宁创造”品牌。扎实推进济宁高新区“人才特区”建设，落实人才优先投入政策，营造创新创业环境，集聚更多高端创新团队和人才，凸显济宁人才优势。充分挖掘和放大文化资源优势，进一步解放文化生产力，发展文化事业，壮大文化产业，改善文化民生，提升文化竞争力和影响力。加强社会主义核心价值体系建设，健全公共文化服务设施，按照“一园三带四区”布局，突破一批关联度高、辐射能力强的综合体项目，推动文化产业集聚集群发展。经过5年努力，使济宁成为创新型经济蓬勃发展、创新人才加速集聚、创新活力充分释放的地区，济宁高新区成为全国重要的技术研发、转化中心，科技进步对经济增长的贡献率提高到55%以上。文化产业占地区生产总值的比重达到5%以上，建成社会风尚良好、人文气息浓郁、文化事业兴盛、文化产业发展的文化强市。

三是优化体制机制、政务服务和社会管理创新“三大环境”。围绕解决全局性、深层次体制机制问题，加大改革攻坚力度，加强统筹设计和整体配套，率先在统筹城乡发展、“两型”社会建设、高效生态经济、农村土地管理等重点领域改革上实现大的突破。深化国有资产管理体制和国有企业改革，鼓励支持民营经济加快发展。统筹推进财税、金融、投融资体制和事业单位改革。实施开放带动战略，构建全方位、多层次、宽领域的对外开放格局，吸引更多战略投资者和社会资本来我市创业发展。着眼建设服务型政府，打造零障碍、低成本、高效率的发展软环境。进一步深化行政审批制度改革，优化再造审批流程，实现行政审批事项全程上网、互联互通，建设“高效政府”；全面推行政务公开，方便群众办事和监督，建设“阳光政府”；加强政府规程建设，坚持科学民主决策，严格依法行政，建设“法治政府”；践行以人为本、民生为先执政理念，落实惠民富民举措，更好地解决就学、就业、就医、养老、住房等民生大事，建设人民满意政府。深入推进社会管理创新，完善社会矛盾疏导和化解机制，形成有效的诉求表达、权益保障和矛盾调处机制。建立社会自治、自律新机制，培育公民意识，强化社区在社会管理中的基础作用。完善网上舆论引导机制，提高对虚拟社会的管理水平。加强社会治安综合治理，健全重大事项社会稳定风险评估机制和应急管理体系，确保“平安济宁”建设取得更大成效，为科学发展跨越发展营造和谐稳定的社会环境。

各位代表，济宁的发展已站在新的历史起点上，把未来五年宏伟蓝图变为现实，离不开全市850万人民的齐心协力、团结实干。我们将凝聚各方智慧力量，不辱使命，勇于担当，不畏艰险，开拓进取，奋力实现建成全面小康社会新目标，向全市人民交出一份满意答卷。

三、2012年重点工作

今年是新一届政府开局之年，也是实施“十二五”规划承上启下的重要一年。我们要围绕主题主线，把握“稳定增势、高位求进、加快发展”工作基调，落实“三个高于、三个提高”目标要求，着力推动工业化、城镇化互动并进，突出投资拉动、项目牵动、开放带动、创新驱动，统筹做好稳增长、控物价、调结构、惠民生、抓改革、促和谐各项工作，实现规模扩大、结构优化、民生改善、社会稳定，开创全市经济社会发展新局面，以优异成绩迎接党的十八大胜利召开。经济社会发展主要预期目标为：地区生产总值增长11%，地方财政收入增长15%，规模以上工业增加值增长15%，全社会固定资产投资增长22%，社会消费品零售总额增长16%以上，外贸进出口增长10%，实际利用外资稳步增加，城镇居民人均可支配收入和农民人均纯收入均增长12%，城镇登记失业率控制在4%以内，居民消费价格涨幅控制在4%左右，全面完成省下达的节能减排约束性目标，人口自然增长率控制在7‰以内。突出抓好十个方面的工作。

（一）做大做强工业经济实力。坚持“双轮驱动”和“两化融合”，完善支持工业发展政策，落实优势产业、骨干企业个性化扶持措施，促使工业结构优化、总量扩充、档次提升。改造升级传统产业，集中推进“千户企业改造提升”、“利税过千万元企业信息化改造”、“创百亿企业”和“百户重点企业扶持”工程。全年技改投入560亿元、占工业投资的比重提高到60%以上。加快建设如意科技180万米高档精纺面料、太阳纸业40万吨高档食品包装卡纸等100个重点技改扩能项目，建成100家信息化改造示范企业、6个“两化融合示范区”，形成百家强企方阵，年内再新增2－3家百亿元大企业。稳定发展能源工业，调整优化电力结构，扶持壮大煤化工、装备制造和食品工业，助推四大千亿级产业及纺织、造纸、建材三大优势产业拉长链条、集群发展、增强竞争实力。强化质量兴市和品牌战略，依托主导产业和骨干企业争创更多驰（著）名商标及名牌产品。培育发展新兴产业，重点在新能源、新材料、生物医药、新一代信息技术和节能环保产业等五大领域建设50个重大项目，加紧实施泰山玻纤3亿米电子布、辰欣药业科技园等系列高端项目，支持冠铨电子、润峰集团健全产业体系，推动太阳能光伏、LED新光源、动力电池及电动汽车、节能环保新产品等加速成长，尽快建成在全国有较大影响力的特色产业链。提升济宁高新区创新发展能力，实施科技服务体系、创新型产业集群建设“双试点”工程，突破重大关键技术，促进战略性新兴产业迅速集聚、高新技术产业快速壮大，增强高新区经济实力和核心竞争力。年内全市高新技术产业产值达到1200亿元，占规模以上工业比重提高1个百分点以上。

（二）大力发展现代农业。继续实施农村六大重点工程，落实强农惠农政策，强化装备技术支撑，大幅提高农业综合生产能力，多渠道增加农民收入。一是调整优化农业结构。毫不放松抓好粮食生产，深入开展高产创建活动，力争全年粮食总产突破100亿斤。落实新一轮“菜篮子”工程和“十大产业振兴规划”，提升大蒜、圆葱、瓜果等优势农产品生产水平，发展壮大食用菌、中药材、苗木花卉等高效产业，扩大畜牧业生态健康养殖和南四湖渔业净水养殖规模，建设特色畜牧业大市和淡水渔业强市。二是提升农业产业化水平。启动农业龙头企业培植提升工程，促使现有骨干企业增量提质，鼓励各类投资者进军农业产业化领域，创建更多基地型、集团化、链条式龙头企业，年内规模以上龙头企业发展到800家。推进农业标准化生产，建成农产品质量安全示范区300处，新增“三品一标”认证100个。加快土地承包经营权流转，鼓励发展特色农业园区和现代农业公司，扩大订单种植，提高农业规模化经营水平。三是强化以水利为重点的农村基础设施建设。基本完成南水北调主体工程建设任务，开工兴建地方配套续建项目，确保2013年顺利通水。推进国家治淮重点工程建设，启动泗河整体改造和引汶入济工程，整治13条中小河流。完成去冬今春农田水利系列项目和9个全国小型农田水利重点县年度建设任务，同步推进田间末级渠系建设。加快构建农村公路网络，新改建农村公路1200公里。抓好农村饮水安全、电网改造，改善农民生产生活条件。农业机械化水平提高2个百分点。四是健全农业服务体系。建立多渠道增加农业投入保障机制，支持引导金融资源向农村延伸，积极发展村镇银行。加快农业科技创新尤其是种业创新，组织科研单位、高等院校和企业开展良种繁育、栽培工艺等农业科技攻关。完成乡镇农技推广、动植物疫病防控、农产品质量安全监管机构改革与建设任务。规范发展专业合作组织，落实新增农业补贴重点向种养大户和专业合作社倾斜的政策措施。搞好农村劳动力就业培训，鼓励农民工外出就业、返乡创业，持续提高财产性和工资性收入比重。

（三）力促服务业跨越发展。进一步创新理念、拓展领域，加快服务业发展步伐，年内服务业投资增长高于固定资产投资增长、增加值增速高于GDP增速。优先发展生产性服务业。紧扣先进制造业加速成长需要，按照社会化服务、专业化分工要求，尽快剥离优势行业中企业非核心业务，推动产前研发、供应和产后销售、服务等业务外包。加紧与知名高校共建工业设计平台，扶持建设20个工业设计创新项目和10家工业设计中心。优化资源配置，组建大型物流集团，引导工业大企业创办第三方物流项目。依托运河“黄金水道”，建设临港物流交易服务中心和京杭运河航道北延及铁水联运港口项目，打响“运河物流”品牌。规划建设金融集聚区，优化金融生态环境，引进民生银行等金融机构入驻济宁，促进证券、保险业健康发展。着力突破文化旅游业。运用市场化理念、专业化队伍、企业化机制吸纳社会资本，深度开发资源，加快建设尼山省级文化旅游度假区等景区景点。完善配套服务设施，推介精品旅游线路，办好“好客山东”贺年会、休闲汇等活动，年内全市旅游社会收入突破330亿元。大力发展生活性服务业。实施新一轮城市商业网点规划，兴建一批各具特色的美食、购物、休闲“一条街”。以太白楼路、金宇路两侧为主体，布局展开大型商业综合体项目，打造中心城区核心商圈。顺应消费层次提升和多元化发展趋势，培育社会化养老、家政服务、医疗保健等消费热点。继续实施“万村千乡”市场工程，扩大农超对接范围，提升配送能力和信息化水平。抓好诚信商贸企业创建工作，健全打击制售假冒伪劣商品工作长效机制，确保群众放心消费。

（四）突出创新驱动引领。一是强化企业创新主体地位。发挥政府引导作用，推动创新资源向企业集聚，创新人才向企业流动，创新政策向企业倾斜。引导企业建立科技投入长效机制，重点企业研发经费支出占比超过5%。支持企业联手大院大所创办高层次研发机构，加紧建设山东科学院济宁分院，确保国家半导体及显示产品质量监督检验中心年内建成运行。全年新增省级以上工程技术研究中心5家、企业技术研发中心6家、院士工作站5家，科技孵化器面积增加到120万平方米。二是加快科技成果培育转化。提升鲁南工程技术研究院、鲁南煤化工研究院、数控技术研发中心等平台装备水平与研发能力，集中突破人才引进、技术攻关、成果转化等关键环节，争取今年引入10个高水平创新团队入驻。实施知识产权战略。办好第十一届中国专博会。建立技术转移承接机制，促进更多创新成果在园区、企业及时转化。年内组织实施省以上科技创新项目200项，培育重大科技成果150项，专利申请量增长20%。三是促进科技金融深度融合。发挥财政资金杠杆作用，创建“天使基金”。吸引境内外股权投资基金、保险基金等机构在我市开展创业投资业务。推进知识产权质押、科技保险、科技担保等工作。启动科技企业上市培育计划，为高成长性科技企业上市融资开辟“绿色通道”。四是壮大高端人才队伍。落实“十二五”人才发展规划纲要，深入实施海外人才引进“511”计划和国内人才集聚“百千万”工程，加强国外智力引进，争取5名以上国家“千人计划”入选者来我市创新创业。以重点行业、骨干企业需求为导向，依托各职业技术院校培养更多技能型、复合型实用人才。优化人才发展机制，营造靠环境吸引人才、靠服务留住人才的良好氛围。

（五）强力突破县域经济。以“大项目突破年”为牵动，纵深推进“县域经济倍增计划”，年内县市区主要指标均达到“三个高于、三个提高”的目标要求。一是培植特色产业。确立“工业强县”理念，增加规模企业数量，促使优势主导产业加速成长。尤其要用好资源禀赋，精心策划特色产业项目，着力拉长农产品加工、装备制造、文化旅游等产业链条，支持有条件的县市区依托现有基础，转化最新科技成果，提升工业经济档次。年内经济强县新上20个、欠发达县新上10个亿元以上产业大项目，每个县市区争取新上几个10亿、50亿甚至超百亿的重量级项目。二是做强经济园区。引导城区搬迁企业、行业技改项目和招商引资项目向园区集中，支持园区特别是14个省级经济园区和6个化工园区增加项目投资密度，加快建成“千亿元”园区，促使优势企业在园区加速成长，重点行业在园区拉长链条，尽快形成骨干企业群体、特色产业聚集区、县域经济增长极。三是振兴民营经济。持续兴起全民创业热潮，支持各类能人特别是科技人才、大学毕业生和返乡农民，带技术带项目带资金自主创业、兴办中小企业。引导现有民营企业调结构、上档次，成长为规模以上骨干企业。建立中小企业公共服务平台，落实“金融助推千家中小企业发展”计划，开展“为企业排忧解难服务活动”，帮助民营企业解决发展中的困难。四是加强财源建设。把做大产业、做优企业与增加财政税收扭在一起抓，夯实财源基础，培植新的财源增长点。抓好项目建设综合效益评估，优先引进和建设高效财源项目。拓宽财源建设领域，大上新兴三产项目，促进现代服务业加速发展，同步放开搞活土地经营和房地产开发市场，加强国有资本收益管理，多渠道增加地方财税收入。坚持依法治税，完善社会综合治税措施，做到应收尽收、足额入库，真正把加快发展的成果反映在财税收入大幅增长上。五是强化政策扶持。落实中央、省扶持政策，研究制定促强扶弱具体措施。对经济薄弱县实施重点帮扶，从税费返还、转移支付、基础设施、项目布局、用地保障等方面给予倾斜。组织强县、大企业开展对口支援，帮助弱县共建经济园区、承接产业转移。

（六）快速提升城镇化水平。以开展“城市建设管理年”为契机，更大力度实施城镇化追赶战略，通过城镇开发建设扩内需、增投资、促发展，年内城镇化率提高2个百分点。

做大做美中心城市。集中突破北湖新区。重点抓好路网完善、设施配套和生态功能组团建设，完成“七纵七横”路网建设任务，确保南二环京杭运河特大桥、北湖连通桥竣工通车，高标准实施北湖湾和核心景区二期工程绿化；加快建设省运会系列场馆，确保省运会指挥中心主体工程年内竣工，建成城市规划展示馆，推进文教、商贸、旅游项目及东西石佛等片区开发，力促北湖新区尽快形成辐射带动能力。提速建设科技新城。密切新城开发与产业发展的有机衔接，全面展开科研工程系列项目，完成科技中心“一体四馆”、大学科技园一期等重大工程，增强高新区吸纳高层人才、研发高端技术、转化高新技术成果的能力和水平。完善济北新区城市功能。完成任城大道两侧产业规划布局，开工建设济宁北二环，抓好高端商务区项目开发，及早实施任城行政中心北迁。高起点改造老城区。启动万达广场等城市综合体项目，实施东南片区城中村及棚户区改造。开工建设金宇路、济安桥路、车站西路改造工程，配套建设供水、供暖、供气及雨污分流等城市基础设施。依靠体制机制创新，精心运作梁济运河、老运河和洸府河“三河六岸”综合改造，加大西跨开发建设力度，促使运河生态新区加速崛起。

推动组群结构城市融合发展。明晰济兖邹曲嘉各大板块功能分区定位，支持各板块以重大基础设施为牵动，以园区建设、产业项目为依托对接融合。推进曲阜高铁片区规划建设，完善高铁站配套交通体系，打造放射状“半小时核心圈”。加快济徐高速济宁段、滨湖路济宁至鱼台段等工程建设进度。提升济宁曲阜机场与日东高速连接线功能。完善兖州至曲阜、邹城城际路网，同步实施公共交通一体化建设，形成“同城效应”。

推进县城和小城镇开发建设。坚持规模扩张与功能提升并重，城镇建设与产业发展齐抓，统筹推进城乡规划、产业发展、基础设施、公共服务、社会事业和生态建设，提升城乡一体化发展水平。鼓励有条件的县改造老城区、拓展新城区、快出新形象。抓好全国重点镇、省级中心镇和新型乡镇建设，培育人口集聚、产业集群和商品集散的特色城镇。尊重农民意愿，有序推进新型农村社区建设。改革户籍制度，支持符合条件的农民进城落户。

提高城市经营管理水平。坚决打破单纯由政府投资搞城建的陈旧模式，建立依靠市场手段聚集社会资本推动城市开发建设的新机制。放开搞活城市建设市场，吸引国内外知名大企业、大财团参与城市开发建设。强化规划执法，坚决查处各类违规违章建筑。建设市级数字化城市管理平台，实施城市管理在线监管。理顺城市管理体制，落实精细化、网格化、动态化管理措施，整治脏乱差，疏解城区交通压力。

（七）进一步深化改革扩大开放。推进国有企业股份制、公司制改革，完善法人治理结构。盘活企业现有存量，做好国有集体困难企业改制重组工作。探索建立省直管县财政管理体制下财权与事权相匹配的市级财政管理新体制。组建市产业发展投资公司和政策性融资担保公司，增强利用财政资金调控和配置社会资本的能力。培育开放的资本市场，吸引各类私募股权投资、风险投资、创业投资机构入驻济宁，引导金融机构和社会资本注入实体经济。探索设立金融租赁、消费金融公司等专业金融服务组织，扩大小额贷款公司覆盖面。健全金融风险预警机制，有效防范和化解金融风险。重视做好企业上市工作，年内争取2－3家企业成功上市。完善市公共资源交易平台，强化交易活动监管。稳步实施事业单位分类改革。深化医疗体制改革，抓好公立医院管理体制、运行机制和补偿机制改革，清理化解基层卫生机构债务。

提升对外开放水平。强化全市开放、全方位开放理念，积极开拓两个市场、吸纳两种资源，借助优质资本、先进管理和技术，兴办高端项目，加快发展步伐。落实部门招商引资责任和任务，形成大招商格局和工作合力。持续兴起招商攻势，组织赴港台、欧美、日韩等重点国家和地区开展境外招商，集中在京津、长三角、珠三角等地开展系列招商活动，加强与世界500强、跨国公司、大型央企的合资合作。把企业推向招商第一线，创新以商招商、产业招商、园区招商方式，争取在现代农业、高端制造业、新兴服务业、节能环保、城市开发等重点领域抓到一批大项目好项目。鼓励大企业特别是驻济省属企业在济宁转型发展、扩大规模，新上项目享受招商引资优惠政策。加强督导考核，加大招商引资在综合考核中的权重，严格依据招商实效兑现奖惩。优化招商环境，健全项目运作“绿色通道”，在环评、审核、立项、供地等环节优化程序、提高效率，确保招商项目依法合规运作落地。严厉打击各种排斥外来竞争、恶意垄断市场的不法行为，保障投资者在我市放心、安心创业发展。落实“境外百展市场开拓计划”，扩大拥有核心技术和自主品牌产品出口，抢占更多市场份额。支持有条件企业“走出去”，打造源自济宁的“跨国公司”。

（八）推动文化大发展大繁荣。组织开展“争创文化强省建设先进市县”活动，落实文化强市建设系列项目和关键举措。从今年起，市财政每年安排文化建设专项资金不少于1个亿。完成经营性文化事业单位特别是一般国有文艺院团、重点新闻网站转企改制任务，推进公益性文化事业单位改革，支持国有文化企业吸引社会资本进行股份制改造。建设和完善公共文化基础设施，加快图书馆、博物馆、文化馆建设，普遍实行公共文化设施向社会免费开放。以第十届中国艺术节在山东举办为契机，繁荣文艺创作，多出精品力作。加强基层文化阵地建设，做好地方特色艺术人才队伍的培养和传承工作，村文化大院、农家书屋年内实现行政村全覆盖。实施文化惠民工程，确保农村公益电影放映一村一月一场、农村公益戏剧演出一村一年一场。推进广播电视由“村村通”向“户户通”延伸，基本实现广播电视人口全覆盖。加快实施孔子文化遗产地保护世行贷款项目，建设孔子博物馆和东方文博城。扎实做好曲阜片区大遗址、南旺枢纽考古和鲁国故城考古遗址公园规划建设及京杭大运河济宁段申遗工作。继续办好孔子文化节、尼山论坛和世界儒学大会。把文化产业作为支柱产业来培植，集中培育10个骨干文化企业、5个重点文化产业项目、5个重点文化产业园区（基地），推进文化产业与旅游、科教、金融深度融合、集群发展。支持有条件的文化企业跨行业兼并重组，培育发展一批传媒、演艺、出版等大型文化企业集团。积极引进国内外优秀文化企业和文化基金，加速文化资源开放性配置和文化要素合理流动。

（九）加快“生态济宁”建设。落实省委、省政府建设“生态山东”重大决策部署，更加注重环保优先、节约优先，走资源节约型、环境友好型发展路子。从严控制高耗能和产能过剩行业扩大规模，坚决淘汰落后产能，严禁新上高排放项目，统筹做好工业、建筑、交通、公共机构等领域节能降耗工作。严格落实“一票否决”和“四不一奖”，对完不成年度节能减排约束性目标的实行项目限批。争取国家、省循环经济和“两型”企业发展基金，力争更多企业（园区）跻身国家级循环经济试点行列。继续对重点行业和企业实行强制性清洁生产审核。落实“治用保防控”举措，“一河一策”制定河流断面整治措施，深化工业点源治理，控制农业面源污染。开展大气污染物监测预警，逐步将PM2.5纳入常规监测体系，消除异味污染。探索采煤塌陷地治理新模式，利用河滩、湖滩和采煤塌陷地规划建设人工湿地。推进环南四湖大生态带建设，实施新一轮林业建设六大工程。强化集约节约用地，严肃查处土地违法行为。

（十）加强以提升民生福祉为重点的社会建设。全面落实中央、省各项惠民政策，继续办好为民承诺10件实事和惠民系列工程。千方百计扩大就业。全年新增城镇就业7万人，农村劳动力转移就业6.8万人，高校毕业生就业率达到85%，确保城镇“零就业家庭”至少1人稳定就业。巩固国家级创业型城市创建成果，开展创业型乡镇创建活动。严格执行最低工资标准，积极构建和谐劳动关系。健全社会保障体系，进一步完善制度提高水平。加大社会保险“一票征缴”力度，继续扩大社会保险覆盖面。巩固城乡居民社会养老保险制度建设成果。按平均10%以上增加企业退休人员基本养老金，对新农合和城镇居民医保补助标准每人每年提高到240元，将城镇居民医疗保险范围内住院报销比例提高到70%，城市低保标准每人每月提高到300－360元，农村低保标准每人每年提高到2000－2400元，农村五保集中和分散供养标准每人每年分别提高到3600元和2100元。落实孤儿等弱势群体福利救助制度，完善社会养老服务体系。加快建设市县残疾人综合服务设施。大规模推进保障性住房建设，开工公租房、经济适用房和廉租房13810套，完成省里下达的年度任务。做好新一轮扶贫开发工作。建立农村贫困家庭危房改造长效机制，让更多农村居民安居乐业。

加快发展社会事业。深入实施教育发展“十二五”规划纲要，保障教育支出依法增长。落实学前三年教育行动计划和学前教育资助政策，完成新改扩建幼儿园100所，学前三年入园率达到78%。抓好普通中小学办学条件标准化建设，加固重建中小学校舍36万平方米。济宁一中新校区实现招生。加强校车安全和校园治安管理。强化高校集群和职业教育基础能力建设，推进职业教育集团化。做好济宁学院上划工作，支持曲阜师范大学、济宁医学院与当地经济社会发展紧密融合，立足济宁扩大规模、提高水平。取消对城乡低保、农村五保对象等困难群众的住院救助病种限制，完善重大疾病专项医疗救助机制。扩大“先看病后付费”就诊范围。启动建设市中医院新院。健全食品安全综合协调机构与风险监测体系，实施放心粮油食品工程，强化粮食行政执法，开展经常性专项整治。加强价格调节基金征收运用，保持市场价格总水平基本稳定。做好23届省运会和第9届省残运会各项筹备工作，深入开展全民健身活动。重视国防动员、人民防空、民兵预备役建设，落实各项优待安置政策，圆满完成征兵任务。稳定低生育水平，改善出生人口性别比。统筹做好民族宗教、外事侨务和对台工作。提升水文、防汛、气象、地震、住房公积金等项工作水平。切实保障妇女儿童、老年人权益。扎实推进对口援疆工作。

加强社会管理创新。完善社会信用体系，打造“诚信济宁”品牌。深化“平安济宁”建设，落实社会维稳第一责任，健全“电子监控全覆盖”安全系统，严密防范和依法打击违法犯罪活动。完善群众诉求表达、社会矛盾调解和社会稳定风险评估机制，有效预防和依法化解社会矛盾。加强应急处置工作，提高防范和应对突发事件的能力水平。强化网络动态管理和网上舆情引导。落实安全生产“一岗双责”，严格执行安全生产隐患“零容忍”和安监执法无障碍制度，严防各类重特大事故发生，巩固发展和谐稳定的良好局面。

各位代表！新形势新任务对政府工作提出更高要求。我们将围绕建设人民满意政府，全面加强自身建设，不断提高服务发展、服务民生的能力和水平。要提升思想境界。自觉把加强学习作为增强本领、履行职责的第一需要，注重政治经济理论学习，加快知识结构更新，用党的最新理论和科学发展创新成果武装头脑、指导实践、推进工作。紧密结合“解放思想跨越发展大讨论”，深入查找影响和制约发展的突出问题，坚决打破一切陈旧模式和条条框框，以全新的理念、高远的境界、战略的眼光，透彻认识发展大势和济宁市情，准确把握内在规律和赶超路径，悉心研究办法对策和推进举措，切实把驾驭复杂局面、领导跨越发展的能力提高到一个新水平。要转变政府职能。更加注重强化社会管理和公共服务，更加注重营造均等环境和增进民生福祉，更加注重服务型和开放型政府建设，进一步精简行政许可与审批事项，实施政企、政事分开，减少政府对微观经济活动的干预。强化政府绩效管理，完善考核体系，对重大部署、重要决策及“三重”任务落实情况实行跟踪问效。严格落实岗位责任制、首问负责制、限时办结制，使政府机构运转更加规范高效。要坚持依法行政。落实《济宁市依法行政“五五”规划》，切实规范行政行为。认真执行人大及其常委会的决议、决定，自觉接受人民政协的民主监督，及时办理人大代表建议和政协提案。贯彻《山东省行政程序规定》，完善公众参与、专家论证、风险评估、合法性审查、集体讨论决定相结合的决策机制。推进政府信息公开，加强电子政务建设，办好“12345”市长公开电话。严格落实行政执法责任制，强化执法监督和责任追究，确保公正、文明执法。要改进工作作风。组织开展“效能提升年”活动，精减会议文件，摆脱繁琐事务，集中精力抓落实、求实效。带头深入改革建设最前沿亲历亲为、靠前指挥，把问题解决在一线、难点突破在一线、任务完成在一线、经验推广在一线。模范遵守党员领导干部廉洁自律各项规定，践行廉政承诺。落实党风廉政建设责任制，完善教育、制度、监督和惩处并重的惩防体系。加强财政资金、重大项目和民生工程的审计监督，支持纪检监察机关依法依纪查办案件，严厉惩治腐败行为，以清廉的政风赢得群众的信任和支持。

各位代表！宏伟的蓝图鼓舞我们开拓奋进，人民的期待激励我们拼搏实干。让我们在中共济宁市委的坚强领导下，团结和依靠全市人民，振奋精神，同心同德，坚定信心，乘势快上，为加快建成全面小康社会而努力奋斗！
